# Nanostructured Lipid Carriers (NLC)-Based Gel Formulations as Etodolac Delivery: From Gel Preparation to Permeation Study

**DOI:** 10.3390/molecules28010235

**Published:** 2022-12-28

**Authors:** Anna Czajkowska-Kośnik, Emilia Szymańska, Katarzyna Winnicka

**Affiliations:** Department of Pharmaceutical Technology, Medical University of Białystok, Mickiewicza 2c, 15-222 Białystok, Poland

**Keywords:** etodolac, nanostructured lipid carriers (NLC), gel formulations, in vitro release, permeation study

## Abstract

Topical administration of drug is an attractive alternative to the oral administration as it provides a reduction in adverse reactions and an enhancement of therapeutic effects. The use of lipid carriers in hydrogel structures makes it possible to introduce lipophilic substances in a dissolved form. In this study, an NSAID from the BCS class II, etodolac (ETD), was used. The nanostructured lipid carriers (NLC) obtained with ETD were incorporated into semi-solid forms (gels). Hydrogels with the suspended drug and oleogel were also prepared for comparison purposes. The obtained gels were tested in terms of pH, viscosity, rheological, mechanical, and bioadhesive properties. The release and permeation through membranes were also studied. All tested formulations were characterized by a pH below 7, which ensured the physiological state of the skin. The viscosities of all gels decreased with increasing shear rate, indicating non-Newtonian behavior. The fastest ETD release was observed for NLC with a Carbopol base (formulation F1); a similar result was noticed in the permeation test. The developed gel formulations containing ETD-NLC dispersion and Carbopol or Poloxamer as gelling agents were stable and possessed beneficial pharmaceutical properties.

## 1. Introduction

Etodolac (ETD), according to the European Pharmocopoeia, is a white or almost white crystalline substance. ETD belongs to the group of non-steroidal anti-inflammatory drugs (NSAIDs), the selective cyclooxygenase-2 inhibitors. Due to its properties, such as anti-inflammatory, analgesic, and antipyretic effects, it is widely used to treat inflammation, especially in rheumatoid diseases. ETD exhibits a relatively short half-life (around 7 h) and requires frequent oral administration, which entails adverse effects (including on the gastrointestinal tract) [1,2]. ETD occurs as a racemic mixture of (+) S and (−) R enantiomers and is classified in class II of the Biopharmaceutical Classification System (BCS) as a substance of poor water solubility (0.016 mg/mL) and high permeability through biological membranes (logP 2.5). ETD is commercially available in oral preparations in the form of tablets or capsules. Apart from one complex preparation containing ETD and other ingredients such as camphor, menthol, and methyl salicylate (Proxym Gel^®^ registered in India), there is no available topical product with this drug [3,4,5,6].

**Figure 1:**
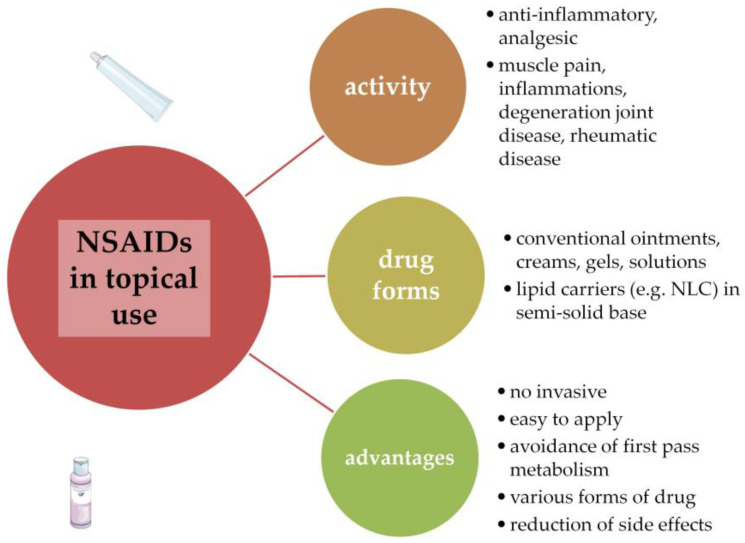
NSAIDs in topical therapy [9,10].

NSAIDs are widely used in the treatment of arthritis in the form of topical applications. Administration of drugs on the skin is an alternative route to oral use and enables patients to avoid side effects and provide simple and comfortable applications. Topical treatment is also an adjunct method for oral administration. The drug effect is stronger, and better results in patient therapy can be achieved. However, topical drug application is associated with drawbacks related to limited skin permeation; therefore, the substance properties and selection of vehicle type affect drug efficacy after skin administration. The conventional topical preparations include semisolid forms (ointments, creams, and gels) and liquid formulations (solutions and emulsions). Innovative ideas concern the use of modern drug carriers incorporated into traditional forms. Among them are the nanolipid carriers—nanostructured lipid carriers (NLC) are one of the novel types of lipid that can be used in topical applications and dissolve the active substance in oil compounds. This approach is especially important for drugs with poor water solubility, which are often associated with limited bioavailability [7,8,9]. Figure 1 shows the use of NSAIDs in topical therapy.

Nanostructured lipid carriers as novel multiunit dosage forms with physicochemical stability, non-toxicity, and the ability to enhance drug absorption are extensively studied as drug vehicles for topical application. NLC are composed of biocompatible liquid and solid lipids dispersed in an aqueous solution enriched with surfactant and/or co-surfactant. The drug is usually dissolved in a liquid oil phase and integrated into solid lipid core. The selection of the compounds impacts NLC pharmaceutical characteristics, including liquid miscibility, drug solubility, drug loading, and drug release profile [11,12,13,14]. Despite their many advantages, the liquid form of NLC limits their topical application. To overcome this challenge, additional semi-solid preparations (ointments, creams, or gels) can be introduced to incorporate NLC structures. For this purpose, a liquid NLC dispersion can be emulsified with the semi-solid base or gelled with polymer (cellulose derivatives, Carbopol, and Poloxamer). Another method is to use the solid form of NLC (obtained in the freeze-drying or spray-drying process) and incorporate it into the base inclusion. The NLC structure in a semi-solid base might significantly affect the properties of topical formulations, thus the rheological, mechanical, and adhesive properties must be carefully evaluated and optimized.

The optimal dermal preparations should enable easy and comfortable application and effective drug action. Gels (hydrogels or oleogels) are a semi-solid alternative to ointments or creams Hydrogels have a three-dimensional structure and have the ability to absorb large amounts of water due to the space between polymer molecules (swelling properties). This semisolid form of preparation is widely used both in the pharmaceutical industry and in cosmetics [15,16,17,18,19,20]. The application of hydrogels instead of traditional ointments or creams has many advantages related to their properties, such as viscoelasticity, flexibility, superabsorbancy, softness, and spreadability. Their hydrophilicity makes application more comfortable because they spread easily on the skin, do not leave a greasy feeling, and can be easily washed off the skin. Biodegradable and biocompatible polymers with bioadhesive properties that enable increased the contact time with the skin surface are particularly attractive in the technology of topical hydrogels [21]. 

Oleogels are lipophilic liquids (natural oils and mineral oils) gelled with suitable substances (e.g., fumed silica, wax, and cholesterol). They are different from hydrogels in their typical lipophilic nature, as they contain no water. Oleogels are used as an alternative to ointments or oily solutions, providing better drug solubility (than ointment bases) and application properties (better spreading on the skin and a less fatty feeling than ointments; they do not run off the skin during application as do the oily solutions). Oleogels do not require the presence of preservatives and are a good carrier for lipophilic substances [22]. In our previous studies, we have recently demonstrated the feasible potential of NLC composed of Capryol 90, glycerol monostearate, and Tween 20 as ETD delivery platforms. By applying an experimental design approach, the technology of NLC was optimized in terms of zeta potential, polidyspersity index, and entrapment efficiency [23]. The aim of the present research was to obtain and characterize ETD-loaded NLC incorporated in hydrogel platforms for topical application. For this purpose, different types of gel-base formulations comprised of Carbopol, xanthan gum, alginate sodium, Poloxamer, and silica were tested. The designed formulations were tested for organoleptic, pH, rheological, mechanical, and bioadhesive properties and compared with gel-base formulations with ETD suspended in a polymer matrix. Particular effort was made toward investigation of the in vitro drug release profile from gel preparations and the ETD permeation pattern through the synthetic membrane and excised human skin.

## 2. Results and Discussion

### 2.1. Preparation and Physicochemical Properties of Gel Formulations

The combination of a lipid carrier (e.g., NLC) and gel in one formulation enables drug dissolution in the oil phase, good biocompatibility with the skin (natural lipids are the composition of carriers), increases drug penetration through the skin membranes, improves occlusive properties (lipids adhere to the skin), and prolongs the release of the drug, and the possibility of incorporation both hydrophilic and lipophilic substances [15,24,25]. NLC form a film on the skin surface and improve the occlusion effect and drug penetration. The composition of NLC—lipids and surfactants—effects skin lipid rearrangement by mixing with stratum corneum lipids, decreases corneocyte packing, disrupts skin structure, and, as a consequence, modifies the therapeutic effect of the drug. The emulsification and ultrasonication method were selected at work to receive NLC dispersions [8,23,26,27,28,29]. Due to its low viscosity and poor adherence upon topical application, liquid NLC was incorporated into semi-solid forms (gel formulations). Appropriate concentrations of gelling agents providing optimal organoleptic and viscosity properties were chosen during the preliminary studies.

In the present study, four gels with NLC carriers (F1–F4), two conventional gels—drugs suspended in bases (F5 and F6)—and one oleogel (F7) were formulated. The process of their preparation included dispersing the appropriate gelling agents in water, liquid NLC, or oil at ambient temperature (F1, F3, F4, F5, F7) or low temperature (F2, F6). The high amount of water in gels required adding preservatives (parabens), and oleogel had vitamin E as an antioxidant substance. Two polymers, Carbopol and Poloxamer, were chosen for the preparation of both NLC-gels and gels with suspended ETD, as they showed more preferred properties than other excipients tested (especially in terms of visually evaluated consistency).

Carbopol is a hydrophilic polyacrylic acid polymer, widely used in the pharmaceutical, cosmetics, and food industries as a thickening, dispersing, emulsifying, rheological, and modification substance. In water, Carbopol forms a colloidal dispersion characterized by low viscosity. The addition of common base (e.g., NaOH, triethanolamine) enables to transformation of the acid polymer into a salt form and increases their viscosity [30,31,32]. Poloxamer (pluronic) is a triblock copolymer (hydrophobic polypropylene oxide flanked by two hydrophilic polyethyleneoxide blocks), a non-ionic surfactant with reversible gelation properties depending on polymer concentration and temperature. In low temperatures, formulations with Poloxamer have a liquid form, while with increasing temperature, their structure changes to a semi-solid (the agelling temperature of a 20% solution is about 20 °C). Pluronic is applied in pharmaceutical formulations as a dispersing, emulsifying, solubilizing, spreading, stabilizing, and wetting agent [32,33,34,35,36]. Alginates are polysaccharides composed of β-D-mannuronic and α-L-guluronic acid units. Sodium alginate, sodium salt of alginic acid, is widely available, cheap to produce, and has benefits such as biocompatibility and nontoxicity, enabling its use in a variety of applications in the food, cosmetics, medical, and pharmaceutical industries (as stabilizing, suspending, viscosity increasing agent, tablet binder and disintegrant, and capsule diluent). Xanthan gum is a high molecular weight polysaccharide gum produced during the fermentation of carbohydrates by *Xanthomonas campestris*. This nontoxic and nonirritant substance is used in food (as a hydrocolloid agent), cosmetics (as a thickening agent), and pharmaceutical products. In oral and topical formulations, xanthan gum acts as a thickening, stabilizing, suspending, gelling, sustained release, and viscosity increasing agent. Aerosil is a fumed silica (colloidal silica and colloidal silicon dioxide) used in oral and topical drugs as an adsorbent, emulsion stabilizer, anticaking agent, suspending agent, and viscosity increasing excipient. Colloidal silica in semi-solid preparations is capable of forming both hydrogels (in concentrations of 15–20%) and oleogels (in concentrations of 5–10%) forms [32,37,38,39].

The properties of formulations administered on the skin play a great role in application features, and they also influence the therapeutic effect (the release of drug from the vehicle). The dermal preparations should be characterized by a pH value below 7 (skin has a slightly acid pH, in the range of 4 to 6), in order to not disturb the skin barrier function, not lead to the skin drying (pH above 7), or increase the sebum production (pH below 4). The viscosity of gels determines the consistency which affects the spreadability of preparation, adherence, and residence to the skin surface. High viscosity can improve adhesion to the skin but can also cause problems during squeezing from the tube, spreading over the skin, and altering the release profile of active substance. The low viscosity preparations have a light consistency and are easy and pleasant to apply, but they may remain poorly on the skin [40,41,42,43].

The pH and viscosity values of all ETD formulations were examined macroscopically (Table 1). The formulations F1–F5 were milky and non-transparent, while F6 and F7 gels were characterized as transparent formulations (Figure S1, Supplementary Materials). All gels were homogeneous, without any signs of phase separation. The pH values of ETD formulations were in the range of 5.9 to 6.9, which indicated their use in dermal application and did not disturb skin barrier functions. 

The viscosity of gel formulations varied between 3.3 and 15.4 Paꞏs, which was the effect of the type and concentration of gelling agents used. The highest viscosity was observed for formulations F2 and F6 containing Poloxamer as a gelling agent (Table 1). The formulation F3 (xanthan gum) displayed the lowest viscosity (3.3 Paꞏs).

### 2.2. Stability of Gel Formulations

The stability experiments were conducted to evaluate the storage conditions—temperature and time. Dermal preparations are usually stored at ambient temperature unless the properties of the active substance or additives require other conditions (e.g., storage in a refrigerator) [40,44]. The prepared gels were stored in tightly closed containers for 60 days in various conditions: 20 °C/60% RH (relative humidity), 40 °C/75% RH, and 4 °C (without humidity control). The changes in pH, viscosity, and organoleptic properties of formulations were assessed after 7, 14, 30, and 60 days. Additionally, after 30 days of storage, all gels were examined in the centrifuge test (stressful conditions for 15 min at a speed of 4000 rpm). 

The significant changes observed during visual observation upon storage were observed for formulations F4 (with sodium alginate) and F7 (with Aerosil). Already within the first 7 days, these formulations exhibited phase separation and color change (Table 2). In the centrifuge test (carried out on the 30th day of storage), formulations F1, F2, F5, and F6 proved be stable (except for F1 which was stored at 40 °C and showed phase separation), while gels F3, F4, and F7 showed phase separation in different storage conditions. 

During the 60 days of storage, no major changes in pH values were observed at all analyzed conditions (value differences were not statistically significant, *p* > 0.05). Only formulations F1, F4, and F5 showed significant differences (*p* < 0.05) during storage at 4 °C and 40 °C (the drop of pH units at 40 °C was about 0.5).

**Figure 2:**
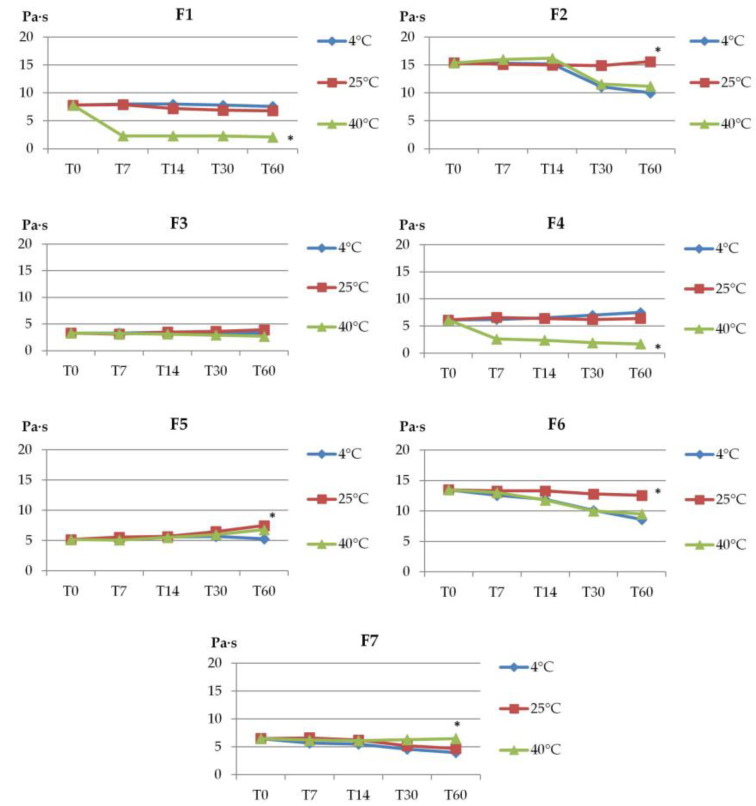
The viscosity of gel formulations during storage at different temperatures (4 °C, 20 °C, 40 °C). Viscosity was measured at 25 °C ± 1 °C and values are expressed as mean ± SD, n = 3; * represents significant differences with *p* < 0.05; (T0—immediately after preparation, T7, T14, T30, and T60—after 7, 14, 30, and 60 days of storage).

Formulations F1, F2, F4, and F6 showed a decrease in viscosity during storage at 40 °C. The highest viscosity decrease was estimated for F1 and F4, from 7.8 to 2.1 Paꞏs and from 6.1 to 1.7 Paꞏs, respectively (Figure 2). At the same time, a nearly two-fold drop in the viscosity of gels F2 and F6 (with Poloxamer) at 4 °C was noticed. In turn, only formulation F3 showed no major changes (*p* > 0.05) in viscosity during storage, regardless of the conditions applied. It should be noted that the tested gels preserved their structure and viscosity upon storage at ambient temperature.

Overall, prepared formulations remained relatively stable in terms of organoleptic properties, except for hydrogels F4 with sodium alginate and F7 with Aerosil, which lost their structures upon 60-day storage. 

### 2.3. The Rheological and Mechanical Properties 

**Figure 3:**
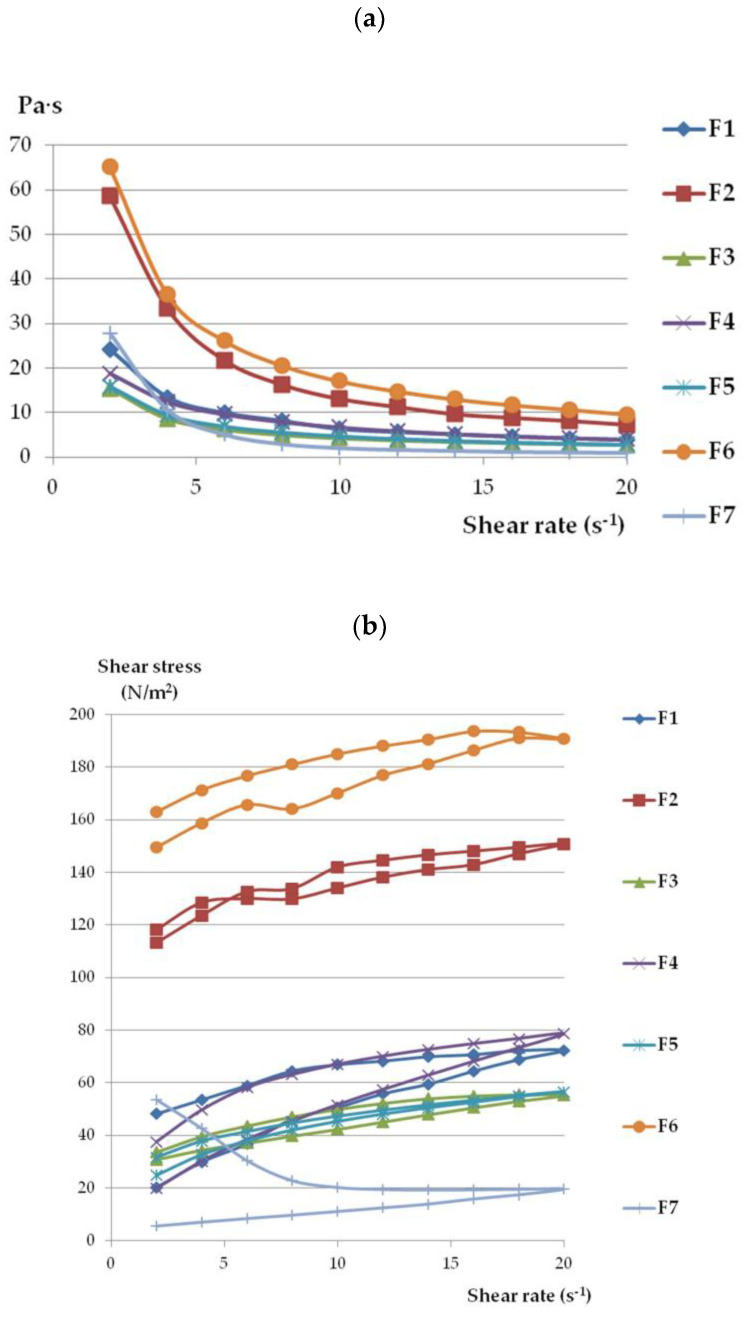
The rheograms (**a**) and flow curves (**b**) of ETD-gels.

The viscosity values of all formulations decreased with increasing shear rate (Figure 3a), which indicated that all gel formulations behaved as shear-thinning, non-Newtonian systems [45,46,47]. The loss in viscosity under increasing shear stress appears beneficial for topical preparations and favors their spreading on the skin surface. As shown in Table 3, the determination coefficients (R^2^) were higher than 0.99 for all gels, confirmig the appropriateness of the power-law model for describing the flow properties of ETD gels. The flow behavior index (n) values were below 0.322, so they are shear thinning systems (viscosity decreases with increasing shear rate) [46]. Both formulations with Poloxamer (F2 and F6) showed the highest viscosity and shear stress values among the tested formulations. 

Based on the hysteresis curves (Figure 3b), it can be seen that formulations F2, F3, and F5 exhibited the highest thixotropic properties because the reconstruction of primary gel structures was achieved (the up curve and down curves are joined at the point of the initial shear rate of 2 s^−1^) Thixotropy refers to the transition of gel to liquid form and the ability to return to the lost phase after time (where shear stress is no longer impacted). This feature appears to be important, particularly during application, because it enables easy spreading of the preparation in contact with the skin by squeezing product out of the tube [44,48]. The slightly weaker thixotropic properties were characterized by formulations F1 (NLC incorporated in Carbopol base), F4 (NCL-gel with sodium alginate), and F6 (ETD suspended in Poloxamer). In turn, oleogel (F7) displayed the lowest thixotropy—10 times lower at the end of the test as compared to initial values. Garg et al. [29] and Han et al. [49] presented data suggesting a correlation between decreased viscosity and increased shear stress in NLC-gels; additionally, the thixotropy features were observed in the gels. Ghica et al.’s [50] work confirmed the thixotropic nature and pseudoplasticity (the non-Newtonian character) of hydrogels.

Hardness and consistency determine the performance of preparations when they are squeezing out of the tube and spreading over the skin surface. The adequate mechanical properties ensure the simple taking of the preparation from the container, facilitate product spreading, and allow the preparation adequate residence time on the skin. Hardness is the highest peak force measured during first compression; it is the maximum force during penetration of the analyzed product. However, the cohesiveness is the area underneath the second compression curve divided by the area underneath the first compression curve and shows how the sample can be deformed [51,52,53]. According to data from Table 4, formulations F1 (NLC with Carbopol), F2 (NLC with Poloxamer), and F6 (ETD suspended in Poloxamer gel) exhibited the highest hardness and consistency values, which corresponded with results from viscosity measurements (Figure 3). The presence of NLC affected the mechanical behavior of gel-formulations. Formulation F1 (NLC incorporated in Carbopol-base) possessed substantially greater values of mechanical properties than gel with suspended ETD (F5)—the values of hardness and consistency were found to be approximately 2-fold higher. This is in accordance with previously published data devoted to the fact that the incorporation of NLC in gels resulted in desirable texture features for topical application [52,54]. Both formulations with Poloxamar—F2 and F6 exhibited the highest texture properties. Interestingly, no impact of the presence of NLC on polymer base mechanical behavior—hardness and cohesiveness—was observed (similar values of analyzed parameters for F2 and F6 gels, *p* > 0.05). Only the consistency parameter was statistically higher for F2 gel than F6 gel (*p* < 0.05). The oleogel (F7) had similar mechanical properties to NLC-gel with a sodium alginate base (F4) (*p* > 0.05).

### 2.4. Bioadhesive Properties

**Figure 4:**
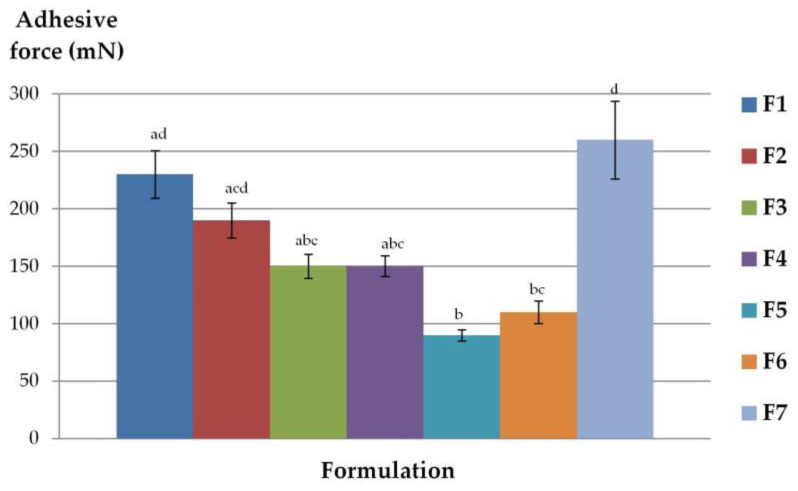
Adhesive force of gel formulations determined using mouse skin (mean ± SD, n = 6). different letters (**a**–**d**) represent the significant differences (*p* < 0.05).

Tensometric studies evaluated the strength required to separate tested gel formulation from the isolated animal skin. Figure 4 displays the maximum detachment force (imitating the mechanical stress resulting from the rapid body movements) and Table 5 shows values of bioadhesion work (imitating the overall capability to remain adhered to the surface of the skin surface upon continuous changing body positions). The higher adhesive force and work is a result of the high adhesion ability and prolonged contact with the biological membrane [55].

Profound differences in ability to interact with animal tissue were noticed between tested formulations. Formulation F7—oleogel with colloidal silica was characterized by the greatest bioadhesive parameters. The high values of adhesive force and work which indicate satisfying adhesion properties possessed also NLC-gels with Poloxamer (F2) and sodium alginate (F4). The lowest adhesive capacity (both in terms of adhesive work and adhesive force) was observed for F5—Carbopol-gel with suspended ETD. It can be assumed that the presence of lipophilic components increased bioadhesion behavior of the gel base, that is the content of the lipid phase are influenced by high adhesion ability. The formulation with the best adhesion properties, F7, contained over 90% of lipid phase (Miglyol) and had high hydrophobic properties. Comparing the formulations where ETD was dissolved in NLC and suspended in the gel base, it can be concluded that the presence of nanolipid carriers enhanced the adhesive properties of gels. It can be especially noticeable for Poloxamer in the assessment of adhesive force and work, where F2 (NLC-gel) and F6 (ETD-suspended gel) exhibited significant differences (*p* < 0.05).

### 2.5. In Vitro ETD Release

The release test for topical preparations determines the capacity of drug diffusion from the semi-solid base to the acceptor fluid (e.g., to the phosphate or the acetate buffers). Factors such as solubility of the drug in the base and the acceptor fluid, drug particle size, oil/water partition coefficient, and rheological properties (especially viscosity) of preparations significantly impact the drug release [48].

**Figure 5:**
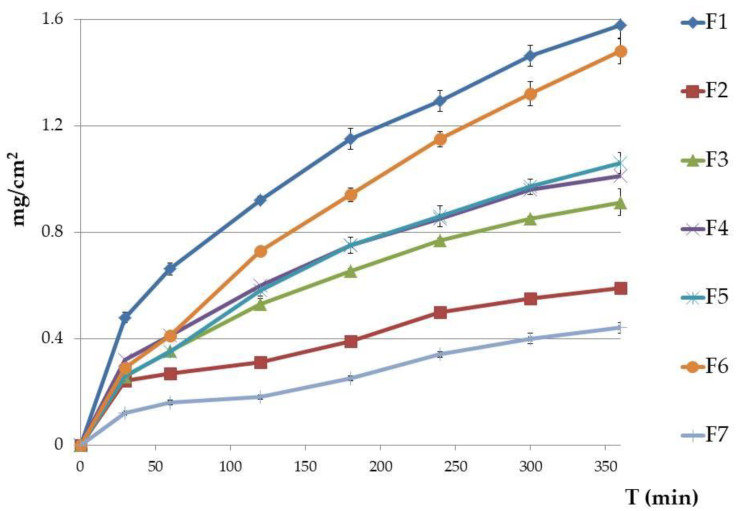
In vitro release of ETD from gel formulations (mean ± SD, n = 6). Results are expressed as the amount (mg) of drug detected in the acceptor fluid, which penetrated the synthetic membrane (Cuprophan) in accordance to 1 cm^2^ of membrane. Formulations F2, F3, F4, F5, and F7 showed significant differences (*p* < 0.005) in comparison to the F1 gel which had the highest amount of ETD release).

ETD release patterns from formulations F1–F7 are presented in Figure 5. After 6 h, the fastest ETD release was noted from formulation F1—NLC-gel with Carbopol. Formulation F6 had only a slightly lower release (difference of 0.1 mg), with Poloxamer and suspended ETD (*p* > 0.05). The lower values were shown for F3, F4, and F5 formulations (0.91, 1.01, and 1.06 mg/cm^2^, respectively), while the smallest amount of ETD released from F2 (NLC-gel with Poloxamer; 0.59 mg/cm^2^) and F7 (oleogel: 0.44 mg/cm^2^). The highest release rate of ETD was recorded during the first 4 h of the test, with ≥80% of the total drug being released and detected in the acceptor fluid.

As ETD is a lipophilic substance (log P 2.5) and has a higher affinity for the oily phase, the slow release profile of ETD from the oleogel formulation (F7) is likely the result of its highly lipophilic nature (oil makes up over 90% of the formulation). Administration of ETD in a hydrophobic preparation can limit drug release from the lipid base to the application place. Therefore, emulsions (especially o/w) and hydrogels that possess greater hydrophilic natures ensure faster release of the drug [56,57].

ETD release from NLC-gel formulations were compared with drug release from NLC dispersions, which was investigated in our previous work [23]. ETD showed lower release from NLC-gel formulations (semi-solid form) than from liquid NLC forms. After 6 h, ETD release from liquid NLC dispersions was above 30% and ranged from 7.5–20% greater than from NLC-gels. This decrease of ETD release is probably a consequence of the gel structure acting as a mechanical barrier to drug release from NLC carriers [29].

To explain the mechanism for ETD release, four kinetic models (zero-order, first-order, Highuchi, and Korsmeyer-Peppas model) were applied. The best fit model was chosen based on the correlation coefficient value (R^2^). Table 6 presents the kinetic analysis of ETD release. The highest R^2^ was obtained for the Higuchi model (R^2^ > 0.96), lower values but close to 0.9 were obtained in the zero-order model. The first-order and Korsmeyer-Peppas models do not fit the mechanism of ETD release from gel formulations, because R^2^ values were below 0.3. Release of ETD according to the Higuchi kinetic model indicates that drug release from gels is controlled by diffusion and could be described as the diffusion process based on Fick’s law—square root of time dependent (Q = kt^0.5^). The Higuchi model is used to describe the release of water soluble drugs, poorly water soluble drugs, semisolid patches, and transdermal patches [58,59].

### 2.6. Permeation of ETD

Penetration studies are considered an essential tool for predicting in vivo topical drug absorption. Although ETD is regarded as a drug with high permeability across biological membranes, there is limited research data devoted to its penetration across the skin barrier [2,30,31,32]. In addition, based on the available literature, NLC may act as a penetration enhancer promoting drug absorption by interacting with lipid bilayer membranes and lipid rearrangements [60,61].

Based on the results from rheological, mechanical, adhesion, and in vitro release studies, for the next step of research permeation tests of the formulations with Carbopol (F1, F5) and Poloxamer (F2, F6) were selected. ETD penetration from Carbopol gel formulations (F1, F5) was investigated using excised human skin. For comparison, in the permeation study the artificial human skin (Strat-M membrane) was also applied. This membrane was used to assess the ETD permeation from formulations: F1, F2, NLC-gels, F5, and F6 gels with suspended ETD. Strat-M is a multi-layered artificial membrane used as an alternative to human and animal skin. This membrane imitates crucial structural and chemical features of human skin. The upper layer of Strat-M is coated with lipids corresponding to those of the stratum corneum and the lower layer simulates epidermis and dermis structures [62].

**Figure 6:**
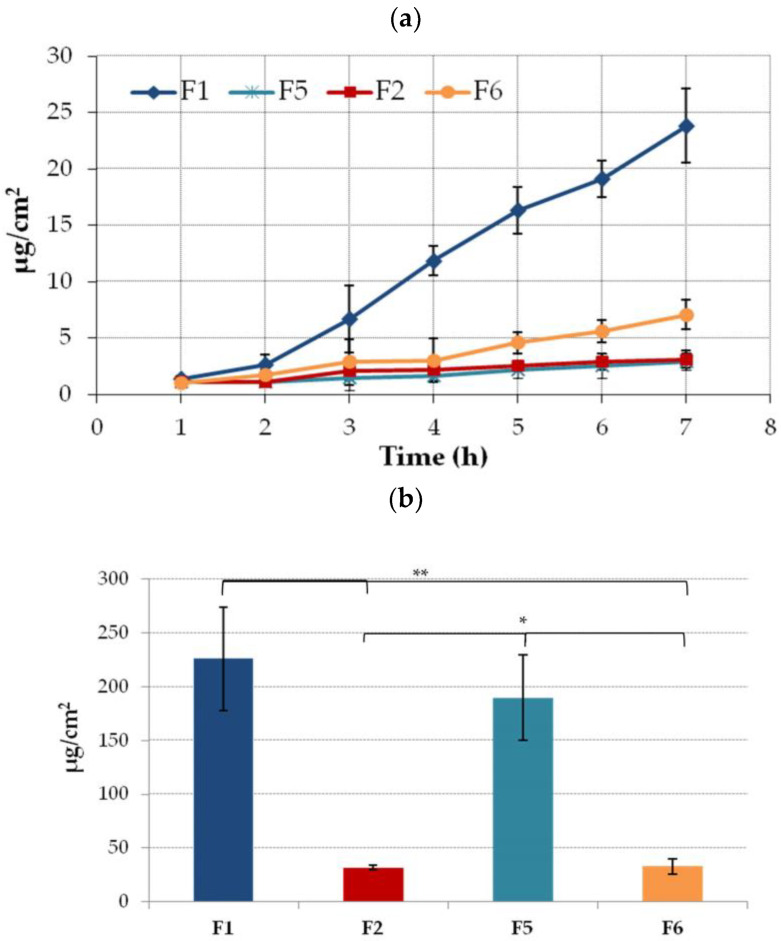
(**a**) Permeation and (**b**) retention (expressed as the amount of drug in the acceptor medium per contact surface area) of ETD encapsulated in the NLC-gel (F1, F2) or ETD suspended in the gel base (F5, F6) through a Strat-M membrane (mean ± SD; n = 4). * and ** represent significant differences with *p* ≤ 0.01 and *p* ≤ 0.001, respectively.

Profound differences in the penetration behavior were noticed between tested formulations. In tests with the Strat-M membrane, relatively low ETD levels were found in the acceptor medium at an early stage of experiment (Figure 6a). After 2h of incubation, the permeated fractions of the drug from formulations F2 and F5 were below 5 µg/cm^2^. The highest amount of permeated drug was observed for formulation F1 comprised of NLC incorporated in Carbopol base. This results correspond to the data from release studies in which the F1 formulation exhibited the highest rate of drug release (Figure 5). In turn, in studies with the F5 formulation, the total amount of ETD that penetrated across the Strat-M membrane after a 7h incubation did not exceed 5 µg/cm^2^. This observation suggests that the presence of NLC in Carbopol base increases ETD permeability. Interestingly, despite profoundly lower values of hardness and consistency of hydrogel F5 in comparison to F1, both drug release (Figure 5) and penetration rates were found to be limited. No substantial differences in ETD penetration behavior during first 4 h of study were noticed between hydrogels F2 and F6 prepared with pluronic. In addition, relatively poor drug absorption through the Strat-M membrane was observed. This could be attributed to the more stiff and solid consistency of these formulations. After putting hydrogels F2 and F6 in contact with thermostated donor compartment, they started solidifying which in turn could limit the drug diffusion rate from the hydrogel carrier. It should be noted that at the end of the test with formulation F6, the permeated fraction of the drug was two-fold higher when compared to formulation F2 containing ETD-loaded NLC. This behavior is consistent with the results from release studies. Surprisingly this is because tween 20 and capryol (ingredients of the NLC dispersion) are considered as penetration enhancers that increase drug absorption [63,64].

The clear impact of the gel base on the ETD accumulation in an artificial skin membrane was observed (Figure 6b). In the studies using hydrogels with Carbopol (F1 and F5), drug retention was significantly higher vs. formulations with a Pluronic base and varied between 226 µg/cm^2^ and 190 µg/cm^2^ for F1 and F5, respectively. The presence of NLC slightly enhanced drug retention.

**Figure 7:**
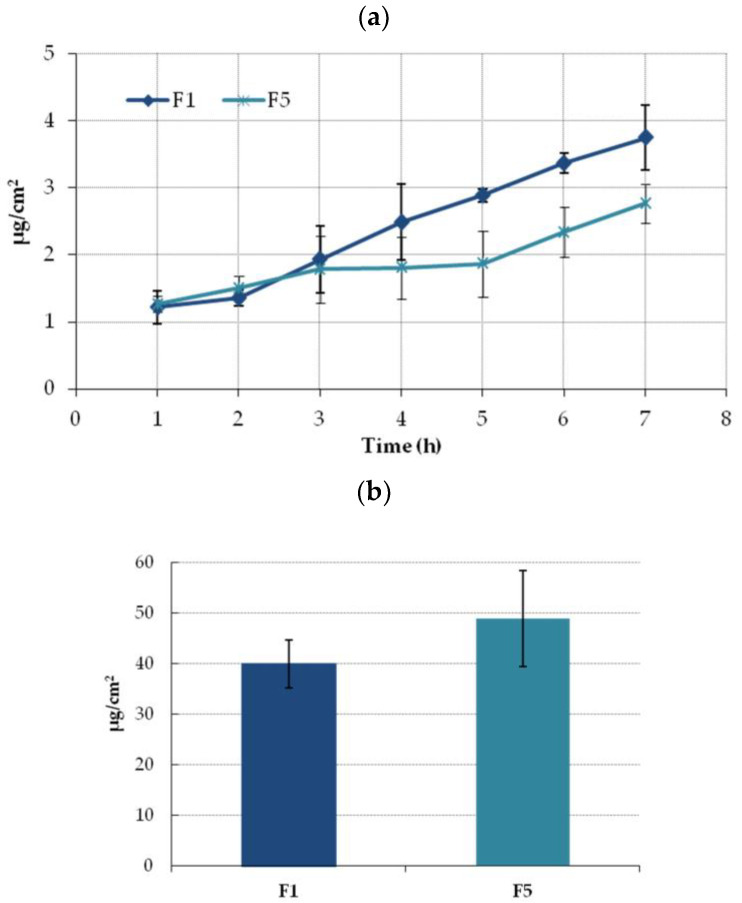
(**a**) Ex vivo permeation and (**b**) retention (expressed as the amount of drug in acceptor medium per contact surface area) of ETD encapsulated in the NLC-gel (F1) or ETD suspended in the gel base (F5) through excised human skin (mean ± SD; n = 4). No statistically significant differences (*p* > 0.05) in ETD retention between F1 and F5 were observed.

As formulations with Carbopol assure greater ETD permeability and retention in the Strat-M membrane, they were further subjected to ex vivo studies with using human excised skin. Figure 7a illustrates the cumulative amount of ETD permeated over time through the human tissue. Similar to the penetration studies with using a Strat-M membrane, comparable fluxes values were noticed between formulations within the first hours of incubation and greater drug permeability was observed in the studies with hydrogel F1. However, at the endpoint of the test, the final ETD concentration in the acceptor fluid for F1 was only 1.5-fold higher than in studies with hydrogel F5. In addition, the drug permeation across human skin was much slower compared with Strat-M membrane. The difference between the two tested membranes based on the higher retention for F1 in Strat-M than in human skin for F5. However, the differences in retention values for formulations F1 and F5 in both membranes were statistically insignificant (*p* > 0.05) (Figure 6b and Figure 7b).

It might be assumed that formulations with Carbopol appear to be a more feasible platform for ETD skin delivery while also ensuring greater drug permeation and retention in the human skin. The incorporation of drug in NLC enhances drug permeability across the membrane when compared to simple suspension of ETD in the Carbopol base. Despite reduced correlation between permeability data attained in tests with excised human skin and artificial Strat-M membranes, this work demonstrated the potential of Strat-M membrane as a screening tool to predict the EDT permeability profile and to select the most promising ETD-loaded formulations for further analysis.

## 3. Materials and Methods

### 3.1. Materials

All chemicals were obtained from commercial sources. ETD was obtained from Xi’an Health Biochemical Technology Co. (Xi’an, China). Capryol 90 (propylene glycol monocaprylate) was supplied by Gattefosse (Nanterre, France). Tween 20 (polyoxyethylene sorbitan monolaurate), Tween 80 (polyoxyethylene sorbitan monooleate), Poloxamer 407 (poly(ethylene glycol)-block-poly(propylene glycol)-block-poly(ethylene glycol), xanthan gum, sodium alginate, and sodium azide were purchased from Sigma Aldrich (Steinheim, Germany). Miglyol 812 (decanoyl- and octanoyl glycerides) was obtained from Caesar and Loretz (Hilden, Germany). Glycerol monostearate was supplied by Paulika (Trąbki Wielkie, Poland), Carbopol 974 by Lubrizol (Brussels, Belgium), and Aerosil 200 (fumed silica) by Evonik (Tokyo, Japan). Triethanolamine was obtained from Stanlab (Lublin, Polska). Methylparaben (4-hydroxybenzoic acid methyl ester) and propylparaben (4-hydroxybenzoic acid propyl ester) were obtained from Pol-Aura (Olsztyn, Poland). Vitamin E was purchased from Hasco-Lek (Wrocław, Poland) and sodium acetate anhydrous from POCH (Gliwice, Poland). Propylene glycol, potassium dihydrogen phosphate, and disodium hydrogen phosphate were provided by Chempur (Piekary Sląskie, Poland). Water was prepared by a Milli-Q Reagent Water System (Millipore, Billerica, MA, USA). Acetonitrile (Merck, Darmstadt, Germany) were of HPLC-grade and other chemicals were of analytical grade.

### 3.2. Analytical Method for ETD Assay

Quantitative analysis of ETD concentration was carried out by high performance liquid chromatography (HPLC) with modifications [65]. In ETD concentration measurement, Agilent Technologies 1260 Infinity equipment (Agilent, Waldbronn, Germany) and Waters Spherisorb ODS2 columns (5 µm, 4.6 × 250 mm; Waters, Milford, MA, USA) were used. The parameters of the method were as follows: the mobile phase was a mixture of acetonitrile and 0.02 M of a phosphate buffer at a pH of 6.0 (in a 50:50 ratio, *v*/*v*) at an isocratic flow (1 mL/min), with the temperature kept at 25 °C. ETD retention time was 3.8 min. The analytical wavelength 225 nm was determined by the ETD UV-Vis spectrum in the length range from 150–400 nm.

The standard stock solution of ETD was prepared by dissolving 10 mg of substance in 10 mL of acetonitrile (1000 µg/mL). Then the appropriate amounts of standard stock solution were transferred to a volumetric flask and diluted by the mobile phase to a concentration in the range of 0.5 to 10 µg/mL.

The linearity of the ETD calibration curve was determined by a linear least squares regression analysis of a plot of peak area vs. ETD concentration. The standard calibration curve was linear ranging from 0.5 to 10 μg/mL (R^2^ = 0.999), and was precise in terms of intra-day and inter-day requirements as reflected by the relative standard deviation values (varying between 0.34 and 2.93% for concentration 5 μg/mL). The limit of quantitation was 0.05 μg/mL. The robustness of the HPLC method was analyzed by small, deliberate changes in the method (changes in pH of the mobile phase, temperature, percentage acetonitrile content, and changes in the wavelength). There were no marked changes (less than 5%) in the ETD chromatograms and the developed HPLC methods were robust.

### 3.3. Preparation of NLC Dispersion

ETD-loaded NLC dispersion consisted of glycerol monostearate as solid lipid, capryol 90 as liquid lipid. Tween 20 as the emulsifying agent was prepared by an emulsification/ultrasonication method previously described by our group [23]. Briefly, an aqueous phase (water, Tween 20, and preservatives) was heated to 70 °C ± 0.5 °C and blended with a hot lipid phase (liquid and solid lipid, ETD) and then mixed (1000 rpm for 10 min). The obtained pre-emulsion was then ultrasonicated (sonicator Vibra Cell VCX500 Sonics and Materials, Newtown, CT, USA) for 9 min at a 40% amplitude and then cooled in an ice bath. The final concentration of components in the NLC dispersion was: 1% for ETD, 4% for the lipid phase (liquid to solid lipid in a 3:7 ratio), 2% for the surfactant, and 0.3% for preservatives (methylparaben to propylparaben in ratio 2:1).

### 3.4. Procedures for Gels Preparation

Selected gelling agents presented in Table 7, Carbopol, Poloxamer, xanthan gum, sodium alginate, and Aerosil (fumed silica) were tested as additives to produce semisolid formulations with ETD. Formulations F1–F4 were obtained using NLC dispersion with dissolved ETD. For comparative purposes, other types of gels were also prepared: F5 and F6 with suspended ETD in the gel base and F7 which included dissolved ETD in the oily phase (Miglyol with Tween 20). Formulations F1–F6 represented hydrogel forms, while F7 was an oleogel (typically lipophilic base).

Gels with NLC carrier were prepared by dispersing the proper amount of gelling agents in a mixture of NLC and propylene glycol (Figure S2, Supplementary Materials). The procedure of dissolving additives in the NLC dispersion was carried out using a mechanical stirrer (600 rpm) at ambient conditions (20 °C ± 2 °C) for Carbopol, xanthan gum, sodium alginate, or in an ice bath (4 °C ± 1 °C) for Poloxamer. The mixing of formulations was continued to complete gelling (Carbopol required the addition of triethanolamine to neutralize the dispersion and gellation).

The procedure for obtaining gels with suspended ETD (Figure S3, Supplementary Materials) included dissolving of the preservatives in hot water (70 °C ± 1 °C) and then cooling of the solution to room temperature. The next step was the gelation of the aqueous solution after adding the polymer (Carbopol at ambient temperature, gelling upon triethanolamine addition; Poloxamer at 4–5 °C). In the final stage, EDT was suspended in the gel base in a mortar (initially preparing the ETD-gel base concentrate at a ratio of 1:1).

The oleogel was also prepared (Figure S4, Supplementary Materials). Firstly, the mixture of Miglyol 812 and Tween 20 was prepared by mixing at high temperature (70 °C ± 1 °C). Then ETD was added, and the mixture was stirred (800 rpm) until the drug dissolved completely. After solubilization, the beaker with oil solution was placed under a mechanical stirrer and silica (Aerosil) was gradually added until gellation. After gelling, vitamin E (antioxidant for the oily base) was added to oleogel and mixed for a short perid of time [22].

### 3.5. Evaluation of Gel Formulations

#### 3.5.1. Visual, pH, Viscosity and Rheological Assessment

All gel formulations were visually evaluated for color, consistency (gelation process) and homogeneity (phase separation). The formulations were assessed immediately after preparation and after 7, 14, 30, and 60 days of storage in various temperature conditions (4 °C ± 0.5 °C, 20 °C ± 5 °C, 40 °C ± 0.5 °C). The pH of tested gels was recorded with an Orion3 pH-meter (Thermo Fisher Scientific, Waltham, MA, USA) at 25 °C ± 1 °C. Analysis of pH in oleogel (F7) was conducted in its aqueous solution (after shaking oleogel with water at a pH 7.0). The viscosity of gel formulations was tested by a cone viscometer (HAAKE Viscotester 6 plus, Thermo Elektron, Karlsruhe, Germany) at a shear rate of 6.00 s^−1^ at 25 °C ± 1 °C. Three repetitions of pH and viscosity tests were carried out and the average values with deviations were calculated.

The rheological tests were carried out using a viscometer HAAKE Viscotester 6 plus at 32 °C ± 1 °C. The measurement was made at increasing and then decreasing the shear rate from 2 s^−1^ to 20 s^−1^. Date were collected and presented as plot of shear stress vs. shear rate (flow curves) and a plot of viscosity vs. shear rate (rheograms). The power-law model to mathematically describe the experimental rheograms (graph of viscosity vs. shear rate) was used, according to the formula:

η = K · γ · n − 1,

where η is the viscosity (Pa·s), K—consistency coefficient (Pa·s), γ—shear rate, n—flow behavior index (power-law index) [66].

For a shear thinning system value of “n” is below 1 (n < 1), the apparent viscosity decreases with increasing shear rate. An “n” above 1 (n > 1) is characteristic for a shear-thickening system. An “n” = 1 indicates Newtonian forms (the shear rate does not influence the viscosity) [45].

#### 3.5.2. Mechanical and Adhesive Properties

The mechanical properties were tested using the texture analyzer TA.XT Plus (Stable Micro System, Godalming, UK), immediately after preparation at room temperature. The disc (35 mm) moved at speed of 1 mm/s and was immersed in the tested gels, while measuring the following mechanical properties: firmness (g), cohesiveness (g × s), and consistency (g).

The texture analyzer was also used to measure the bioadhesion of the tested formulations. In the study, we used the hairless mouse skin (Cby.Cg-Foxn1nu/cmdb mouse strain). The skin samples were obtained from animals intended for organs harvesting, and this study did not require the approval of the local ethical committee for animal experiments. Before analysis the skin samples were thawed (stored at −20 °C), cut into appropriate pieces and kept in 0.9% natrium chloride solution. The piece of skin was attached to the lower end of the measuring probe, whereas the tested gel was located below (0.5 g gel was placed on gelatin disc). Adhesion measurements between the skin and formulations were carried out at 32 °C ± 0.5 °C, using a 0.5 N force for 60 s. Bioadhesive properties were determined as the adhesive force (mN) and adhesive work (µJ). All measurements (mechanical and adhesive) were carried out six times and the average values were calculated.

#### 3.5.3. Stability Studies

All formulations were stored in tightly sealed containers which protected them from light, humidity, and contamination. The gels were stored at three temperature conditions: 4 °C ± 0.5 °C (fridge), 20 °C ± 5 °C (60% ± 5% RH), and in a climatic chamber at 40 °C ± 0.5 °C (75% ± 5% RH). The stability test was fulfilled after 7, 14, 30, and 60 storage days by visual observation, pH measurement, and viscosity measurement. The stability assessment by centrifugation (for 15 min at 4000 rpm) was carried out after 30 days.

#### 3.5.4. In Vitro Drug Release Study

The in vitro release study was performed in an Agilent 708-DS apparatus (Agilent Technologies, CA, USA) using a Cuprophan dialysis membrane (regenerated cellulose, Medicell, London, UK). The acceptor fluid was an acetate buffer (pH 5.5) with the addition of 1% Tween 80 to maintain *sink* conditions. The use of a buffer with a pH of 5.5 imitated the mildly acidic surface of natural skin. The enhancer cell (3 g of gel was located in the chamber covered with a cellulose membrane) was placed in a beaker filled with 100 mL of buffer (32 °C ± 0.5 °C) and at predetermined time intervals (from 30 min to 6 h) the acceptor samples were collected, filtered, and then analyzed by HPLC (described in Section 3.2). The ETD release study was performed in six repetitions.

#### 3.5.5. Drug Permeation Study

##### Excised Human Skin

Freshly excised human skin was obtained from an Noviline Surgery and Aesthetic Medicine Clinic in Bialystok (according to bioethical permission number R-I-002/305/2019) from women (30–40 year-old) undergoing face lifts. Directly after the surgery, skin specimens were preserved in an isotonic saline solution and frozen at −20 °C for no longer than 30 days. Prior to experiments, tissue samples were gently thawed at ambient conditions, cut into pieces (with 2 mm thickness), and checked microscopically for tissue integrity.

##### In Vitro Penetration Studies

In vitro penetration studies were carried out in a flow through cell system equipped with thermostated teflon Bronaugh diffusion chambers and peristaltic pomp (MCP Process IP 65, Ismatec, Wertheim, Germany) according to previously described methods [67]. Two permeability models were applied: artificial membrane Strat-M (Strat-M^®^ membrane, 25 mm, Merck, Darmstadt, Germany) with structural resemblance to human skin and excised human skin received during face lift surgery [68]. Based on the data from rheological, mechanical, adhesive, and release studies, four hydrogel formulations prepared from Carbopol (F1, F5) and Poloxamer (F2, F6) containing ETD-loaded NLC (F1, F2) and suspended ETD (F5, F6) were selected for the penetration studies.

Excised human skin or artificial Strat-M membranes were placed in line diffusion chambers with a contact diffusion area 0.81 cm^2^, and proper amounts of formulations (corresponded to 5 mg of ETD) was applied on their surface. Chambers were thermostated at 32 °C ± 1 °C and compartments with formulations remained closed throughout the experiments. ETD is characterized by pKa value of 4.65, therefore a pH greater than the pKa is charged and exhibits better solubility [69]. In penetration studies 10 mL of a phosphate buffer (pH 7.4with addition of 0.001% sodium azide) was used as an acceptor phase. The medium was flown beneath the skin or artificial membrane in a close-loop system at a constant rate of 40 mL/h. At determined time intervals, samples of acceptor medium were withdrawn, filtered, and analyzed for ETD content by HPLC (described in Section 3.2). The samples were replaced by a same volume of acceptor phase.

At the end of the studies, hydrogels from the donor compartment were aspirated into glass flasks and the skin or membrane surface was carefully washed 20 times with phosphate buffer (pH 7.4). To evaluate drug retention, artificial membrane was immersed in 10 mL of the mobile phase (consisting of acetonitrile and phosphate buffer with a pH of 6.0, in a ratio of 50:50), sonicated (5 min at 30 °C), and incubated at 30 °C for 5 h in water bath with continuous shaking (150 rpm). In turn, skin samples were initially homogenized to form a suspension in phosphate buffer, which was then diluted with the mobile phase up to 10 mL and incubated for 5 h at 30 °C. The extract was filtered through a 0.2 µm nylon filters and analyzed by HPLC method. Penetration and retention behavior was expressed as the amount of ETD permeated to acceptor medium per skin or membrane’s unit area. All tests were performed at least in four replicates.

### 3.6. Statistical Analysis

The test results are presented as mean ± standard deviation (mean ± SD). The statistical analysis was performed using the Statistica 13.3 software (StatSoft, Kraków, Poland). The differences between formulations have been assessed by a one-way ANOVA, with a post-hoc Tukey’s test. The statistical significance level was set at less than 0.05 (*p* < 0.05).

## 4. Conclusions

The selection of the best gelling agents to obtain ETD-gels was performed. Important parameters determining the application of gels is their stability during storage and administration. Formulations with Carbopol (F1, F5) and Poloxamer (F2, F6) proved to be stable during 60 days of storage at 20 °C (60% ± 5% RH). All formulations showed favorable thixotropy properties and were characterized as the non-Newtonian system. Both formulations with Poloxamer (F2 and F6) showed the highest viscosity, while formulations F1 (NLC with Carbopol), F2 (NLC with Poloxamer), and F6 (ETD suspended in Poloxamer gel) exhibited the highest hardness and consistency. Oleogel with silica (F7), NLC-gels with Poloxamer (F2) and sodium alginate (F4) were characterized by the greatest bioadhesive parameters, probably as a consequence of the presence of lipophilic components. The fastest ETD release was noticed for formulation F1—NLC with a Carbopol base—a similar effect was observed in the permeation test with the Strat-M membrane and human skin. It can be stated that developed NLC-gels containing NLC dispersion with dissolved ETD and gelling agents—Carbopol and Poloxamer—enable to obtain stable semisolid formulations, characterized by beneficial pharmaceutical properties.

## Figures and Tables

**Table 1:** Visual observation, pH, and viscosity of the gel formulations (mean ± SD, n = 3).

Formulation	Visually Observation	pH	Viscosity (Pa·s) *
F1	milky, non-transparent, homogeneous gel	6.9 ± 0.03	7.8 ± 0.2 ^a^
F2	milky, non-transparent, homogeneous gel	6.6 ± 0.04	15.4 ± 0.0 ^b^
F3	milky, non-transparent, homogeneous gel	5.9 ± 0.04	3.3 ± 0.1 ^c^
F4	milky, non-transparent, homogeneous gel non	6.0 ± 0.01	6.1 ± 0.3 ^d^
F5	milky, non-transparent, homogeneous gel	6.5 ± 0.01	5.2 ± 0.2 ^e^
F6	transparent homogeneous gel	6.1 ± 0.01	13.5 ± 0.1 ^f^
F7	transparent homogeneous gel	6.3 ± 0.01	6.5 ± 0.0 ^dg^

* different letters (^a–g^) in the same column present the significant differences (*p* < 0.05).

**Table 2:** Visual observation and centrifuge test during stability evaluation.

Formulation	Visually Observation (During 60 Days of Storage)	Centrifuge Test (After 30 Days of Storage)
F1	apperance unchanged	stable at 4 °C and 25 °C, phase separation at 40 °C
F2	appearance unchanged	stable at all temperatures
F3	appearance unchanged	phase separation at all temperatures
F4	color change to brown-cream, separation of water on the gel surface	phase separation at all temperatures
F5	appearance unchanged	stable at all temperatures
F6	appearance unchanged	stable at all temperatures
F7	color change to yellow, separation of oil on the gel surface	phase separation at all temperatures

**Table 3:** Mathematical analysis of the power-law model.

Formulation	K *	N **	R^2^
F1	41.4	0.20	0.998
F2	113.0	0.08	0.998
F3	24.1	0.26	0.995
F4	31.7	0.31	0.997
F5	26.5	0.25	1.000
F6	115.2	0.17	0.999
F7	75.4	−0.50	0.991

* K—consistency coefficient (Pa·s); ** n—flow behavior index.

**Table 4:** Textural properties of gel formulations (mean ± SD, n = 6).

Formulation	Hardness (g) *	Cohesiveness (g) *	Consistency (g × s) *
F1	96.8 ± 0.6 ^a^	37.4 ± 1.1 ^a^	190.5 ± 3.7 ^a^
F2	101.9 ± 1.5 ^b^	79.2 ± 1.3 ^b^	175.1 ± 7.5 ^b^
F3	36.8 ± 0.7 ^c^	13.5 ± 0.2 ^c^	81.9 ± 1.3 ^c^
F4	30.2 ± 0.4 ^d^	12.9 ± 0.1 ^cd^	59.0 ± 0.1 ^d^
F5	43.4 ± 1.8 ^e^	21.0 ± 1.1 ^e^	88.4 ± 4.6 ^ce^
F6	98.1 ± 2.5 ^abf^	81.1 ± 2.1 ^bf^	132.2 ± 1.4 ^f^
F7	27.9 ± 0.5 ^dg^	12.1 ± 0.2 ^cdg^	59.5 ± 0.5 ^dg^

* different letters (^a–g^) in the same column present the significant differences (*p* < 0.05).

**Table 5:** Adhesive work of formulations determined using hairless mouse skin (mean ± SD, n = 6).

Formulation	Adhesive Work (µJ)
F1	363.3 ± 54.2 ^a^
F2	27,950.9 ± 232.7 ^b^
F3	201.5 ± 5.7 ^a^
F4	19,225.6 ± 326.1 ^c^
F5	148.8 ± 19.5 ^a^
F6	497.6 ± 15.8 ^a^
F7	37,607.5 ± 2036.2 ^d^

Different letters (^a–d^) indicate significant differences (*p* < 0.05).

**Table 6:** Kinetic model of ETD release from designed gel formulations.

Formulation	Kinetic Model				
	Zero-Order		Higuchi		
	R^2^	K_0_	R^2^	K_H_	LT (h^1/2^)
F1	0.914	0.005	0.999	0.105	0.15
F2	0.894	0.002	0.975	0.038	0.82
F3	0.925	0.003	0.999	0.062	0.18
F4	0.912	0.003	0.998	0.069	0.14
F5	0.945	0.003	0.994	0.073	0.71
F6	0.973	0.005	0.983	0.102	1.39
F7	0.960	0.001	0.963	0.029	0.82

K_0_—the zero-order proportional constant; K_H_—the Higuchi dissolution constant; LT—lag time.

**Table 7:** Composition of ETD-loaded gel formulations (all gels containing ETD at concentration of 1%).

Formulation	Gelling Agent	Composition
**F1** NLC-gel	Carbopol	NLC dispersion with Carbopol (0.5%), propylene glycol (10%) and triethanolamine (1%)
**F2** NLC-gel	Poloxamer	NLC dispersion with Poloxamer (15%) and propylene glycol (10%)
**F3** NLC-gel	Xanthan gum	NLC dispersion with xanthan gum (1%) and propylene glycol (10%)
**F4** NLC-gel	Sodium alginate	NLC dispersion with sodium alginate (1.5%) and propylene glycol (10%)
**F5** gel with suspended ETD	Carbopol	Carbopol (0.5%), propylene glycol (10%), methylparaben (0.2%), propylparaben (0.1%), triethanolamine (1%), water
**F6** gel with suspended ETD	Poloxamer	Poloxamer (15%), propylene glycol (10%), methylparaben (0.2%), propylparaben (0.1%), water
**F7** gel with suspended ETD	Aerosil	Aerosil (5%), Tween 20 (1%), vitamin E (0.01%), Miglyol 812

## Data Availability

The data supporting the reported results are available on request from the corresponding author.

## Supplementary Materials

The following supporting information can be downloaded at: https://www.mdpi.com/article/10.3390/molecules28010235/s1, Figure S1: Visual observation of the gel formulations. Figure S2: Technology of NLC-gel (F1–F4) preparation; Figure S3: Technology of gels with suspended ETD (F5, F6) preparation; Figure S4: Technology of oleogel (F7) preparation.
