# Supplementary material for: Nanostructured Lipid Carriers (NLC)-Based Gel Formulations as Etodolac Delivery: From Gel Preparation to Permeation Study
Source: Molecules. 2022 Dec 28;28(1):235. doi: 10.3390/molecules28010235 (PMC9821982; doi:10.3390/molecules28010235)
Supplement: Supplementary file 1 [file molecules-28-00235-s001.zip › molecules-2118026-Supplementary Materials.pdf]

molecules

Supplementary Materials: Nanostructured Lipid Carriers (NLC)-Based Gel Formulations as Etodolac Delivery: From Gel Preparation to Permeation Study

Anna Czajkowska-Kośnik, Emilia Szymańska, Katarzyna Winnicka

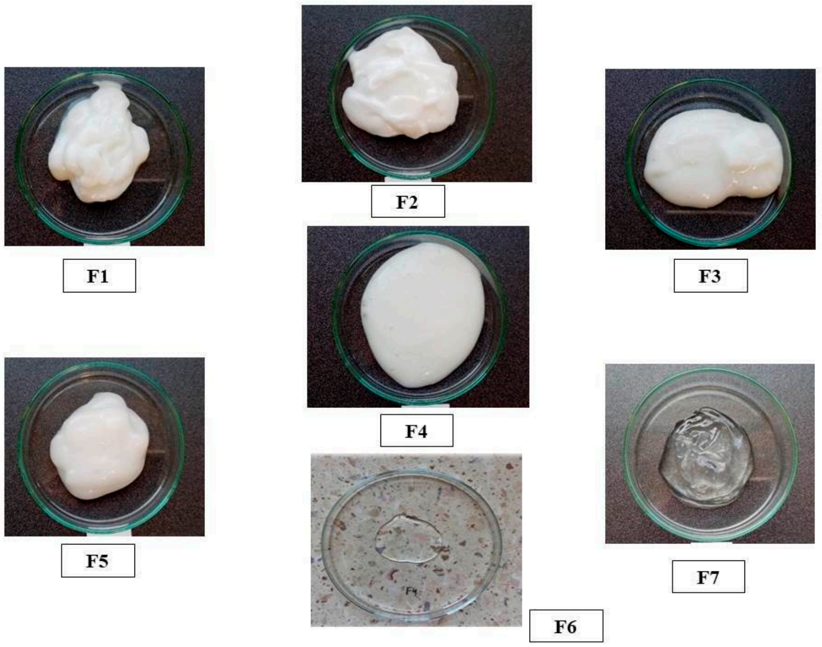

Figure S1. Visual observation of the gel formulations.

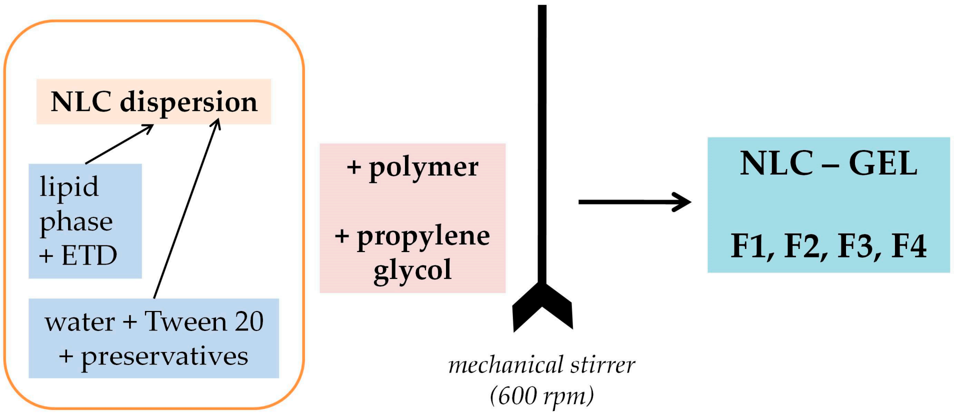

Figure S2. Technology of NLC-gels (F1- F4) preparation.

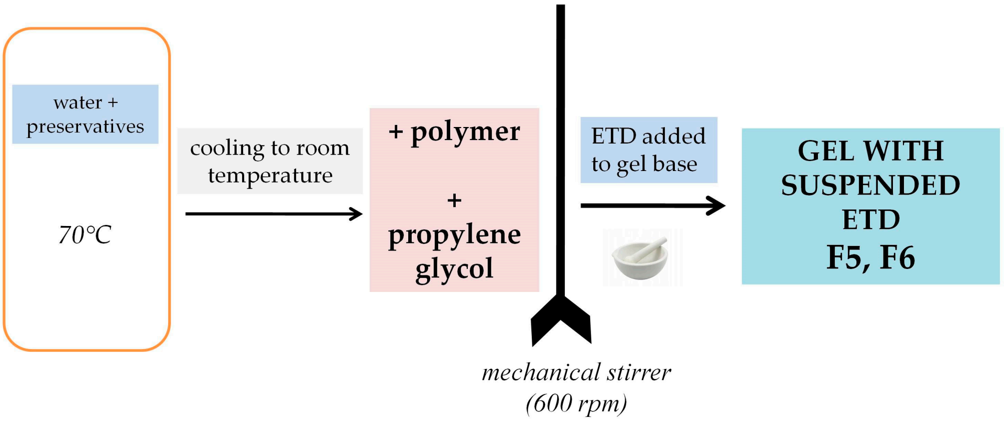

Figure S3. Technology of gels with suspended ETD (F5, F6) preparation.

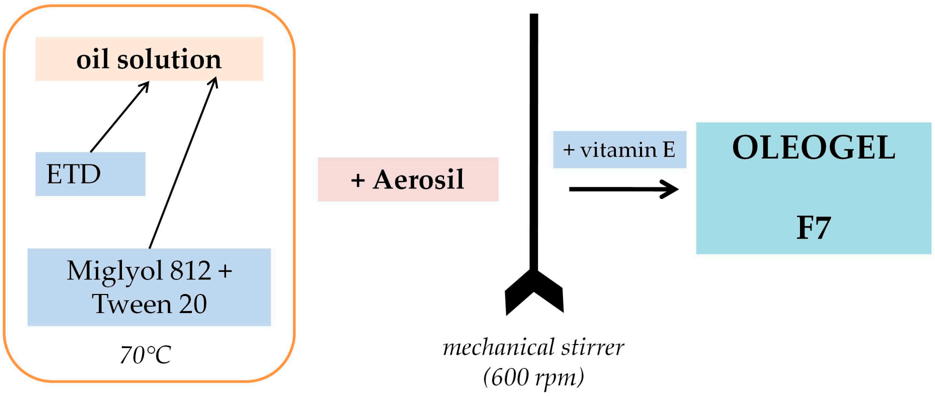

Figure S4. Technology of oleogel (F7) preparation.
