# Supplementary figures and images for: Nanostructured Lipid Carriers (NLC)-Based Gel Formulations as Etodolac Delivery: From Gel Preparation to Permeation Study
Source: Molecules. 2022 Dec 28;28(1):235. doi: 10.3390/molecules28010235 (PMC9821982; doi:10.3390/molecules28010235)

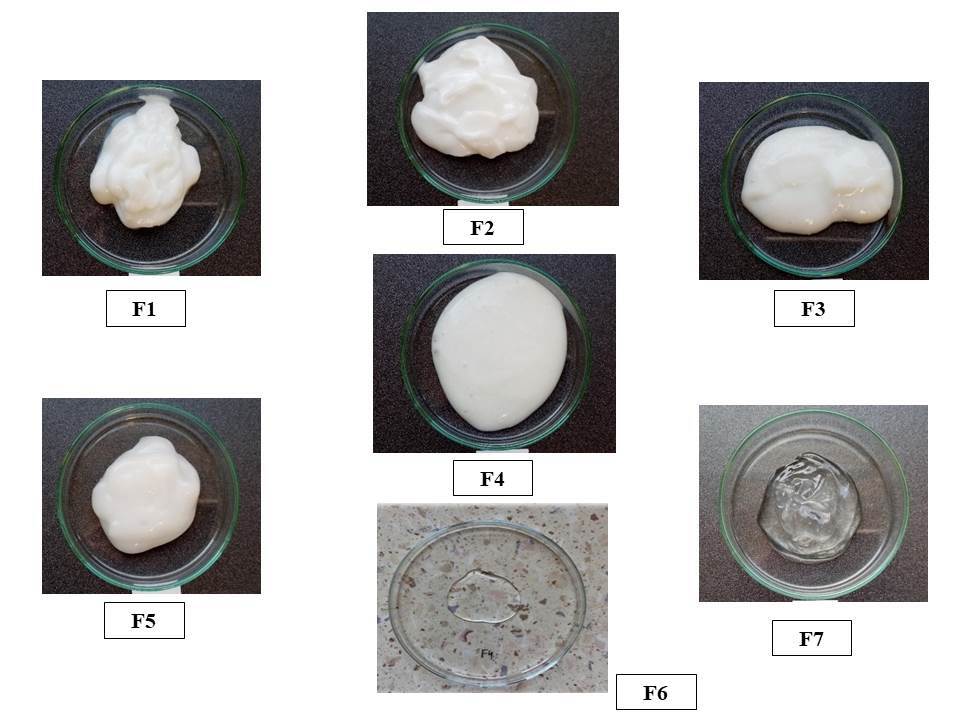

Supplement: Supplementary file 1 [file molecules-28-00235-s001.zip › Figure S1.jpg]

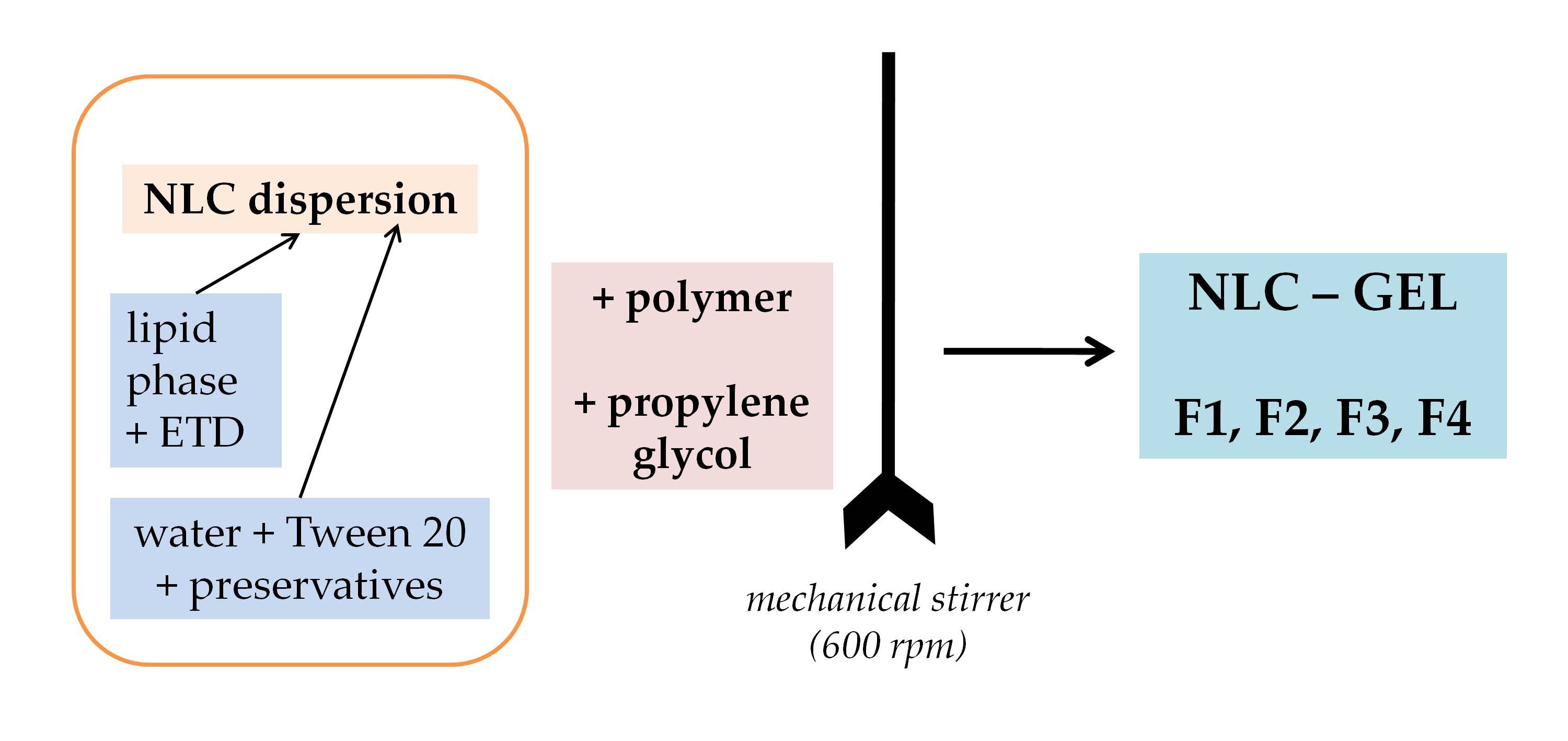

Supplement: Supplementary file 1 [file molecules-28-00235-s001.zip › Figure S2.jpg]

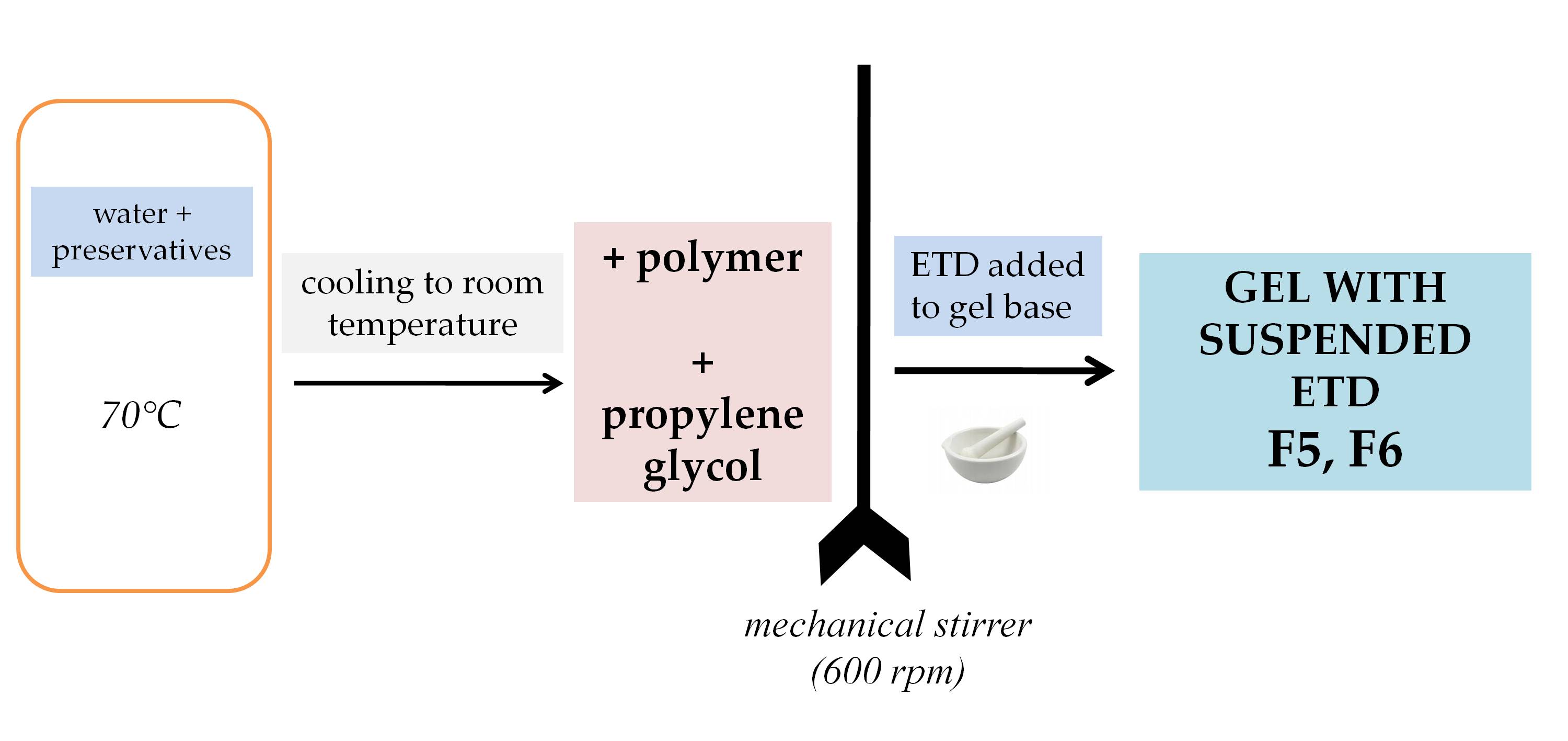

Supplement: Supplementary file 1 [file molecules-28-00235-s001.zip › Figure S3.jpg]

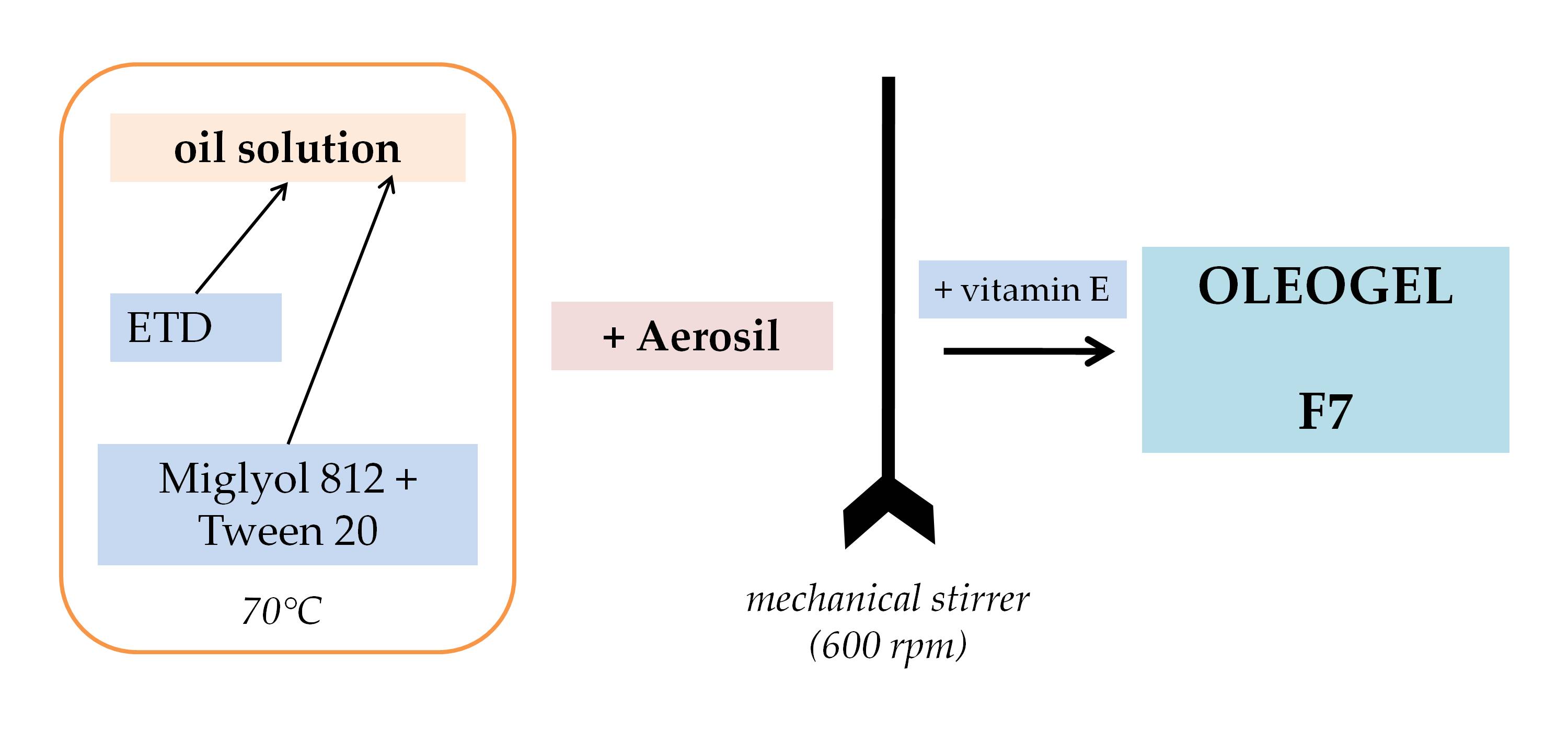

Supplement: Supplementary file 1 [file molecules-28-00235-s001.zip › Figure S4.jpg]
